# Supplementary material for: Enhancing Selective Antimicrobial and Antibiofilm Activities of Melittin through 6-Aminohexanoic Acid Substitution
Source: Biomolecules. 2024 Jun 14;14(6):699. doi: 10.3390/biom14060699 (PMC11201590; doi:10.3390/biom14060699)
Supplement: Supplementary file 1 [file biomolecules-14-00699-s001.zip › biomolecules-3046039-supplementary.pdf]

Supplementary Data

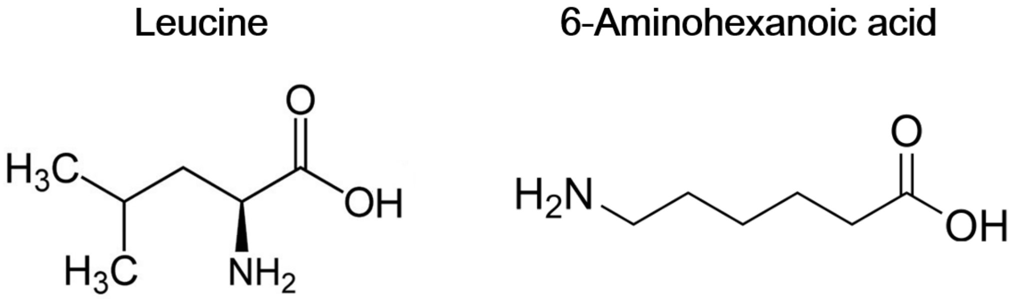

Figure S1: Chemical structures of leucine and 6-aminohexanoic acid. Despite having the same molecular weight as leucine, 6-aminohexanoic acid provides increased flexibility and mobility thanks to its central aliphatic chain.

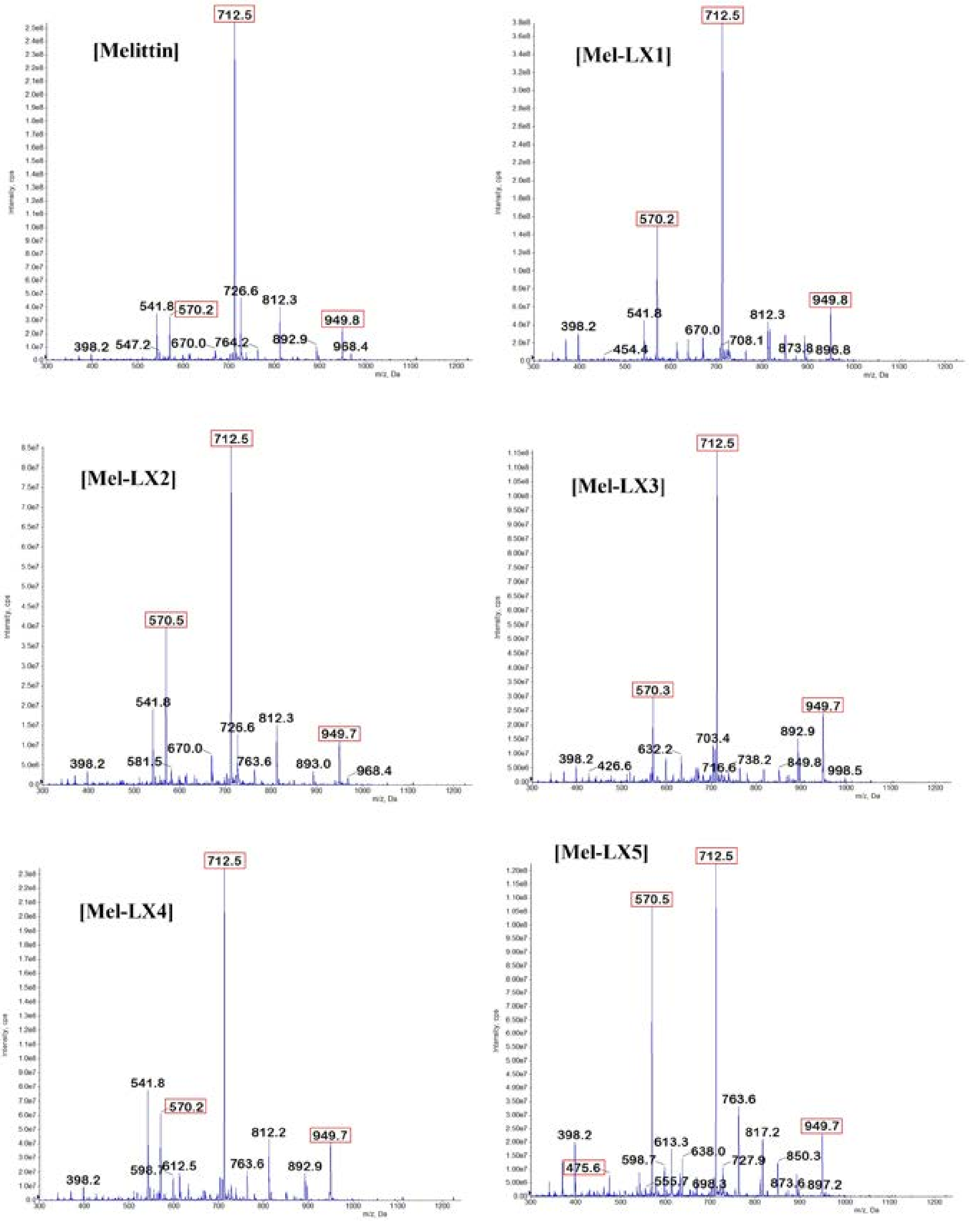

Figure S2: ESI-MS spectra of peptides. The molecular masses of peptides were analyzed using Electrospray Ionization-Mass Spectrometry (ESI-MS).

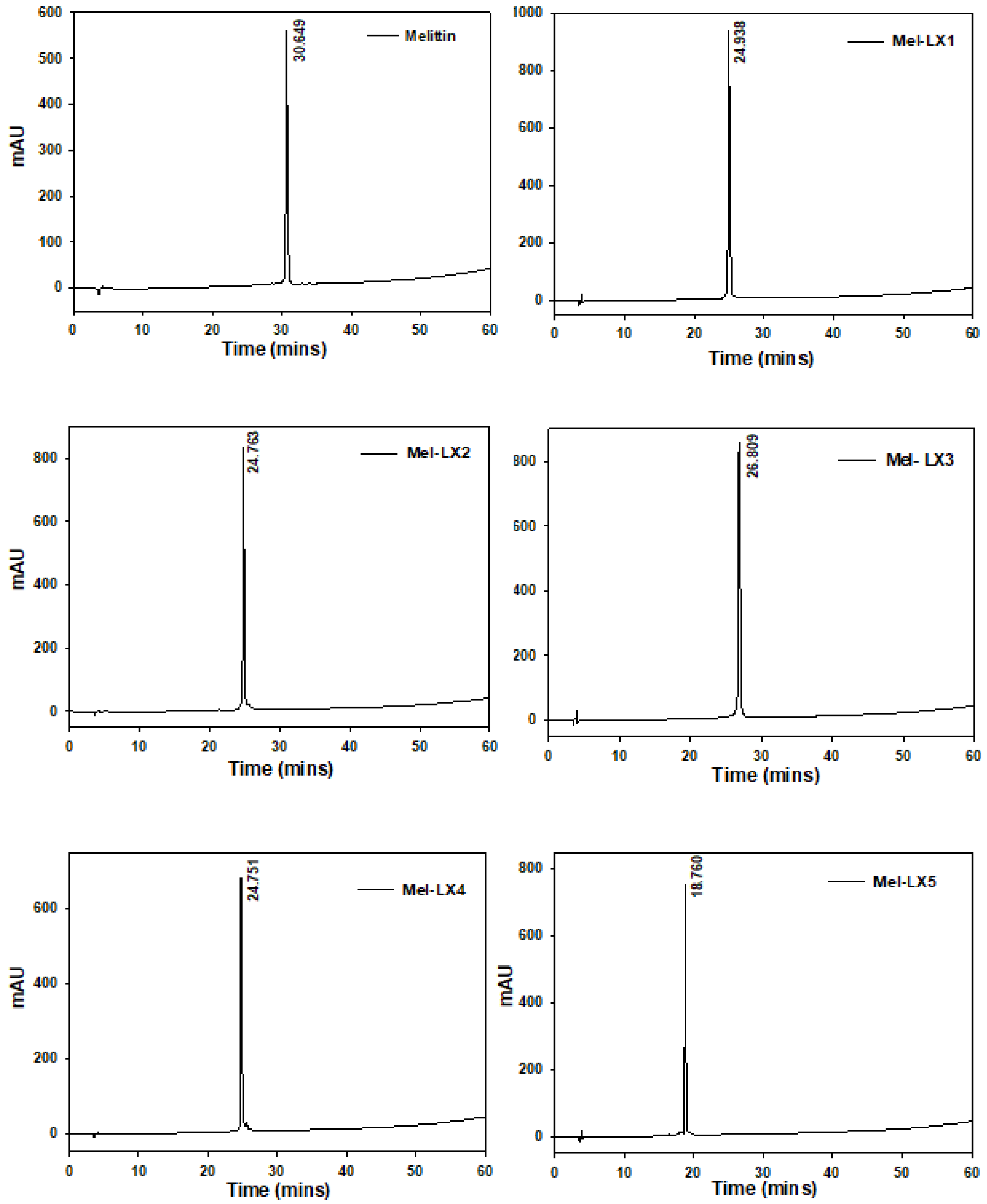

Figure S3: Analytical RP-HPLC profiles of peptides. Peptides were eluted for 60 min with a flow rate of 1.0 mL/min by analytical RP-HPLC on a C_{18} column (5 mm; 4.6 mm × 250 mm; Vydac) using a gradient of buffer B (0.05% TFA in CH_3CN/H_2O 90:10 v/v) in buffer A (0.05 %TFA in H_2O).
